# Boric Acid Affects the Expression of DNA Double-Strand Break Repair Factors in A549 Cells and A549 Cancer Stem Cells: An In Vitro Study

**DOI:** 10.1007/s12011-024-04082-y

**Published:** 2024-02-17

**Authors:** Tuğba Semerci Sevimli, Aynaz Ghorbani, Bahar Demir Cevizlidere, Burcugül Altuğ, Murat Sevimli

**Affiliations:** 1 Cellular Therapy and Stem Cell Production, Application, and Research Center (ESTEM), Eskişehir Osmangazi University, Eskişehir, 26040 Turkey; 2 Department of Histology and Embryology, Faculty of Medicine, Eskisehir Osmangazi University, 26040 Eskişehir, Turkey

**Keywords:** Lung cancer stem cell, Boric acid, DNA DSB, Cytotoxicity

## Abstract

DNA double-strand break (DSB) repair genes interact with tumor stemness- and resistance-associated processes in cancer stem cells (CSCs). Therefore, targeting DNA DSB genes in cancer treatment is important for the CSC phenotype. Although the anti-cancer effect of boric acid (BA) has been studied, its effect on DNA DSB is unclear. Moreover, no studies investigate BA’s effects on DNA DSB of lung cancer stem cells (LC-SCs). To fill the gap, we aimed to assess the effects of BA on A549 cancer stem cells. CSCs were isolated from human non-small cell lung cancer cells (A549) and characterized by flow cytometry. Different concentrations of BA (at doses ranging from 1 to 100 mM) were applied to cancer stem cells. Cytotoxic activities were determined using the cell viability assay (MTT assay) at 24 and 48 h. Expression levels of DNA DSB genes that BRCA1, BRCA2, RAD51, KU70/80, ATM, and XRCC4 were evaluated by RT-qPCR. Additionally, immunofluorescence staining analysis was exploited for caspase-3 and E-cadherin. ATM expression increased significantly (*p* < 0.001). No significant change was observed in the expression of other genes. Moreover, BA up-regulated caspase-3 and E-cadherin expression. Consequently, we can say that BA affects DNA DSB and the apoptotic abilities of LC-SCs.

**Graphical Abstract:**

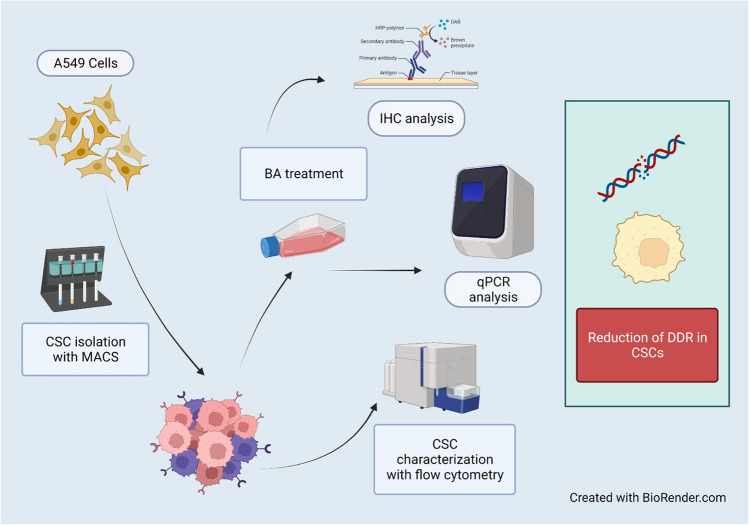

## Introduction

Lung cancer is the leading cause of cancer deaths worldwide. Since it is often not diagnosed until an advanced stage, the mortality rate is relatively high [1]. Detailed pathogenesis is very important for early diagnosis and treatment of lung cancer. In particular, the identification of new biomarkers is necessary for the screening of high-risk populations (e.g., smokers, those exposed to smoke, oil fields, toxic occupational sites). Correct diagnosis is vital for treating lung cancer patients most appropriately [2]. Lung cancer is histologically divided into two types: small-cell lung cancer (SCLC) and non-small cell lung cancer (NSCLC). The overall 5-year survival rate of lung cancer, including SCLC and NSCLC, is the lowest of all cancers [3]. Therefore, there is an urgent need to identify sensitive and specific biomarkers for early diagnosis.

Cancer stem cells, defined by stem-like features with high self-renewal and differentiation capacity, are responsible for the growth and spread of the disease [4, 5]. Lung cancer stem cells have been shown to derive from various cell sources. In particular, it makes cells expressing the cancer stem cell marker glycoprotein prominin-1 (CD133) highly resistant to proliferative and metastatic potential [6, 7]. Identifying CSCs in lung tumors provides a focal point for various possible treatments and therapies, specifically targeting stem-like cells. Therapeutic targets for CSCs include regulating Wnt, Hedgehog, and Notch signaling cascades essential for metastasis [8, 9]. These signaling pathways play vital roles in lung development and regulation of stem cell self-renewal. They may initiate tumorigenesis when mutated, causing dysregulation of the stem cell renewal process and directed and appropriate differentiation [10, 11]. Drug resistance in cancer stem cells (CSCs) may arise from inherent or acquired genetic/epigenetic changes and external factors mediated by the microenvironment and the stem cell niche. Intrinsically, mechanisms of drug resistance in CSCs involve heightened expression of detoxifying enzymes and drug transporters, epigenetic remodeling that supports stemness pathways, metabolic reprogramming, induction of epithelial-mesenchymal transition (EMT), improved DNA repair mechanisms, and induction of dormancy [12, 13].

DNA DSB repair mechanism has diverse biological functions beyond DNA damage response (DDR) signaling, influencing the regulation of CSC survival and therapy resistance [14]. Boron, a non-metallic element, is naturally present in the forms of borax (sodium tetraborate; Bx) and boric acid (BA), with the latter being the prevalent boron form found in plasma [15]. Boron has been found to impact the antioxidant defense mechanism by decreasing the activity of enzymes responsible for inflammation and restraining the proliferation of cancer cells, suggesting the potential clinical utility of boron-containing compounds. Boron derivatives and boric acid demonstrated cytotoxic effects on the colon [16], ovarian [17], lung cancer [18, 19], hepatocellular [20], and prostate cancer [21] cells.

In this study, we aimed to show the effect of boric acid, which we previously showed on MCF-7-derived breast cancer stem cells, on A549-derived cancer stem cells [22]. After BA application to A549 isolated CSCs, DNA Double Break Repair gene expression levels were analyzed by RT-qPCR. In addition, our study is the first in the literature.

## Materials and Methods

### Cell Culture

A549 (human lung carcinoma, CCL-185) cell line was purchased commercially from the American Type Culture Collection. A549 cells were cultured as adherent cells in RPMI-1640 (Capricorn Scientific, RPMI-A) medium containing 10% FBS (GIBCO, cat. no 16000044) and 1% penicillin–streptomycin (Sigma-Aldrich, cat. no P4333-100ML). A549-CSCs were isolated by magnetic cell separation (MACS) technique using monoclonal antibodies against CD117, CD44, CD338 and CD133. Spheres were cultivated in Tumorsphere Medium XF (PromoCell, cat. no C-28070). The cells were cultured at 37 °C under 5% CO_2_.

### Cell Viability

The cells were seeded in the 96-well plates with a volume of 50 µL and a density of 1 × 10^3^ cells. Concentrations of BA (Sigma, cat. no B6768-5006) solutions (ranging from 1 to 100 mM) were administered to the cells using a culture medium devoid of FBS. According to our previous studies, these concentrations were selected [22, 23]. The plates were then placed in a 5% CO_2_ incubator at 37 °C under 95% relative humidity for 24 and 48 h. Following the incubation periods, each well-received MTT solution (Invitrogen, cat.no M6494) was left to incubate for 2–4 h. Afterward, the medium was aspirated, and DMSO solution (Invitrogen, cat.no D12345) was added in low-light conditions. Subsequently, the plates were subjected to analysis at a wavelength of 540 nm using a microplate reader (Biotek ELx808IU, USA).

### Flow Cytometry

A549 cancer stem cells were exposed to FITC-conjugated monoclonal antibodies targeting CD133 (Biolegend, cat. no 393906), CD338 (Biolegend, cat. no 332020), CD44 (Biolegend, cat. no 338806) and CD117 (Biolegend, cat. no 313216) surface markers, along with appropriate isotype controls, in the dark at room temperature for 45 min. Subsequently, cells were washed with PBS (Capricorn, cat. no PBS-1A) solution containing 0.1% sodium azide, followed by centrifugation at 1300 rpm for 5 min. The cell suspensions were analyzed using the AGILENT NovoCyte D3005. The data were processed using custom software for analysis.

### Reverse Transcription-Polymerase Chain Reaction Assay

Total RNA extraction was initially conducted using the RNA isolation kit (RNeasy Micro Kit, Qiagen, 74,004) to determine gene expression levels. Subsequently, cDNAs were synthesized from the obtained RNAs using the cDNA synthesis kit (Qiagen, 330,404). For the real-time PCR reaction, a master mix solution containing SYBR Green (Qiagen, cat. no 330501) and gene-specific primers for BRCA1, BRCA2, RAD51, KU70/80, ATM, and XRCC4 (Table 1) was prepared [22]. Analyses were performed utilizing the Rotorgene Q5 plex + HRM Real-Time PCR, and data  were acquired through the Rotor gene Q software program. Changes in gene expression levels were assessed using the 2^−ΔΔCt^ method.

**Table 1:** Primer sequences used for RT-qPCR analysis [22]

Gene	Primer sequences (forward, reverse)
GAPDH	CACCCTGTTGCTGTAGCCATATTC GACATCAAGAAGGTGGTGAAGCAG
BRCA1	ATCATTCACCCTTGGCACA CATGGAAGCCATTGTCCTCT
BRCA2	CCTGATGCCTGTACACCTCTT GCAGGCCGAGTACTGTTAGC
ATM	TGATAGTAGTGTTAGTGATGCAAACG CAGCTAAAGGATTAATGGCACCT
RAD51	TGAGGGTACCTTTAGGCCAGA CACTGCCAGAGAGACCATACC
KU70	AGAGTGAAGATGAGTTGACACCTTT CCAAGAGATCTCGATCACTGCT
KU80	CCCAAATCCTCGATTTCAGA CCCGGGGATGTAAAGCTC
XRCC4	CTTGGGACAGAACCTAAAATGG GACGTCTCAGGTAGTGAAGAATCA

### Immunofluorescent Analysis

For this assay, cells were seeded in 24-well plates with coverslips at a density of 0.5 × 10^5^ cells/well. Once the cells adhered, they were treated with BA solution for 24 and 48 h. Following incubation, cells were fixed with methanol, washed with PBS, and incubated in PBS containing 1.5% normal blocking serum for 30 min. Subsequently, they were exposed to the primary antibody, diluted in Antibody Diluent solution, at room temperature for 2 h. After 3x wash with PBS for 2 min each, cells underwent a 30-min incubation with labeled secondary antibodies for immunofluorescence studies at room temperature. Finally, samples were covered with a closing medium containing DAPI (UltraCruz Mounting Medium for fluorescence with DAPI), and images were captured using a fluorescent microscope (Leica DMI 4000 Microsystems). 

### Statistical Analysis

Each experiment was repeated at least three times. Obtained data were evaluated as mean ± SD and assessed with Student’s *t*-test and Tukey test. The significance level *p* < 0.05 was considered statistically significant for the differences between the study and control groups.

## Results

### A549-CSCs Express CD44, CD133, CD338, and CD117

**Fig. 1:**
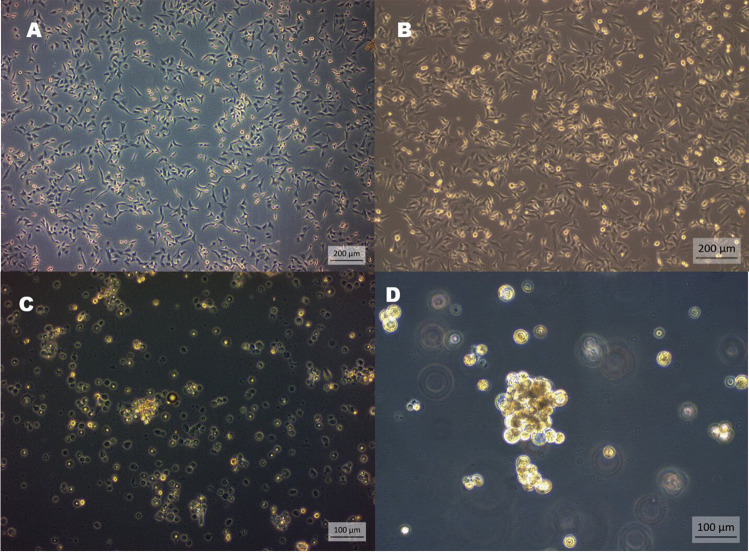
The morphology of A549 (human non-small lung cancer cells) and A549 derived cancer stem cells (LC-SCs). **A**: A549 cells (scale bar = 200 µm); **B–C**: LC-SCs (scale bar = 200 µm and scale bar = 100 µm, respectively); **D**: Tumorspheres formation of LC-SCs (scale bar = 100 µm)

**Fig. 2:**
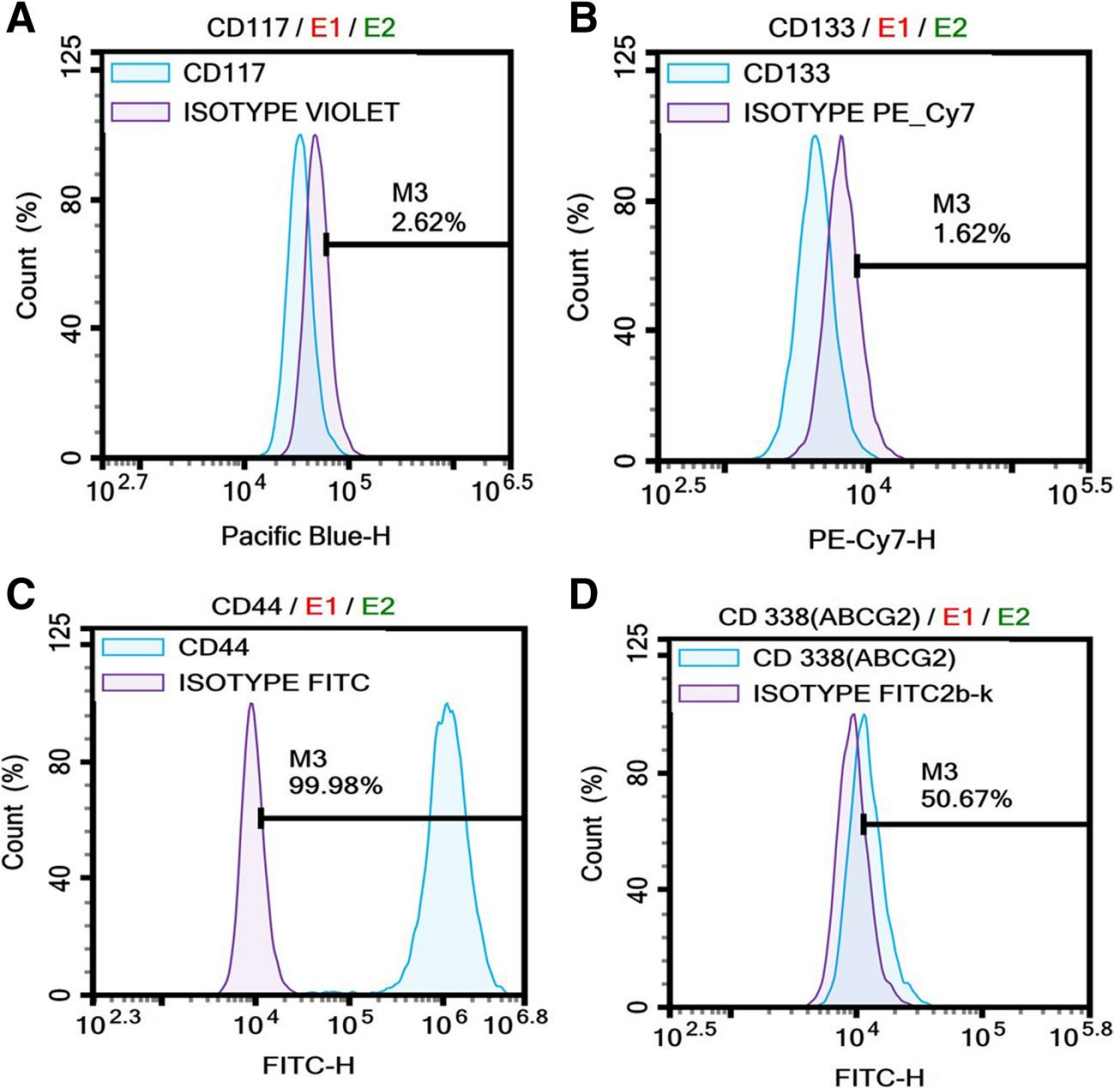
Identification of LC-SCs by flow cytometry. Cell surface expression of CD44, CD133, CD117, and CD338 in the A549 cell line. Flow cytometry analysis detected CD44, CD133, CD117, and CD338 cell populations

Adhesive characteristics were observed in A549 and A549 cancer stem cells under monolayer culture conditions (Fig. 1A, B, and C). A549-CSCs exhibited the formation of spheroids with suitable shape and size (Fig. 1D). Flow cytometry analysis examined CD44 +, CD133 +, CD338 +, and CD117 + cells within the isolated cell population. The results in Fig. 2A–D revealed the presence of CD117 +, CD133 + , CD44 +, and CD338 + cell populations, respectively.

### BA Reduces the Growth of A549 Cells/Spheroids

**Fig. 3:**
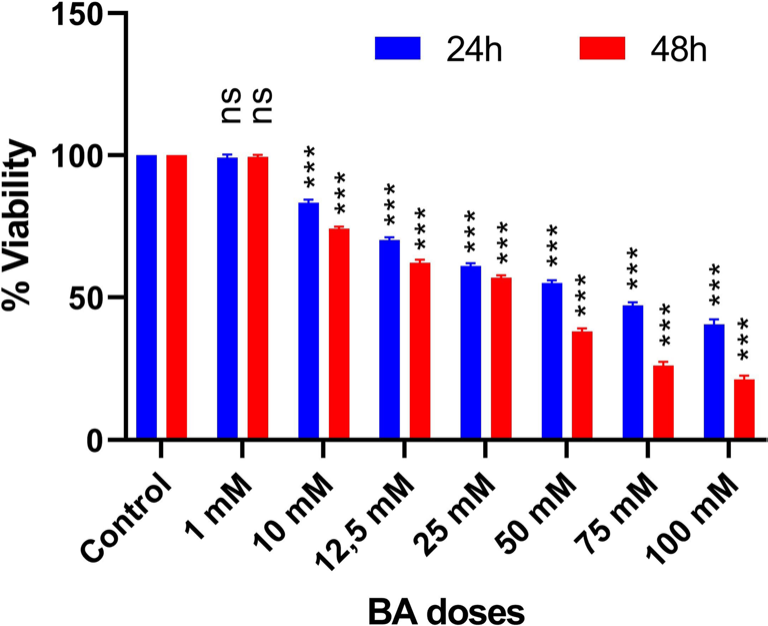
Cell viability of LC-SCs. Cell viability was measured with the MTT assay. LC-SCs were exposed to different concentrations of BA (1, 10, 12.5, 25, 50, 75, and 100 mM) for 24 and 48 h. (BA, boric acid). (mean ± SD; *n* = 3; *P < 0.05, **P < 0.01, and ***P < 0.001; ns means not significant)

In the analysis with A549-CSCs, no difference was observed in cell viability between BA and control groups at 1 mM dose. In contrast, at other doses, it was observed that the viability of BA groups decreased significantly (all *p* < 0.005) (Fig. 3). Cell viability analysis revealed a significant decline in cell viability between 10 and 100 mM doses).

### Inhibitory Potency of BA on the Relative Expression of DNA Double-Strand Break Repair Genes in A549-CSCs

**Fig. 4:**
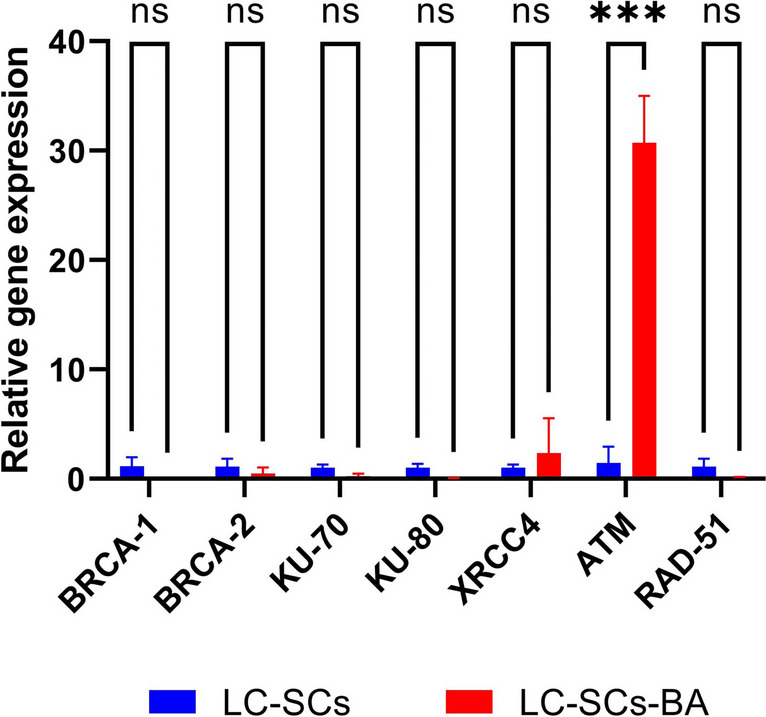
mRNA expressions of DNA DSB repair genes. RT-qPCR was performed as described in the previous study [22]. GAPDH was used as a control. ATM expression increased significantly (*p* < 0.001). (mean ± SD; *n* = 3; *P < 0.05, **P < 0.01, and ***P < 0.001; ns means not significant)

In our previous study [22], we investigated the impact of boric acid (BA) on the expression of DNA double-strand break (DSB) repair genes in breast cancer stem cells. Despite applying the same doses, while BRCA1 and BRCA2 were up-regulated, the expression of ATM (*p* < 0.001), RAD51 (*p* < 0.001), and KU70 (*p* < 0.001) was downregulated in BC-SCs treated with the specified doses (*p* < 0.001). In the present study, the expression of ATM upregulation creased approximately 30-fold in A549-CSCs after BA treatment (Fig. 4). There was no significant change in the expression of other genes (Fig. 4).

### Caspase-3 and E-Cadherin Analysis

**Fig. 5:**
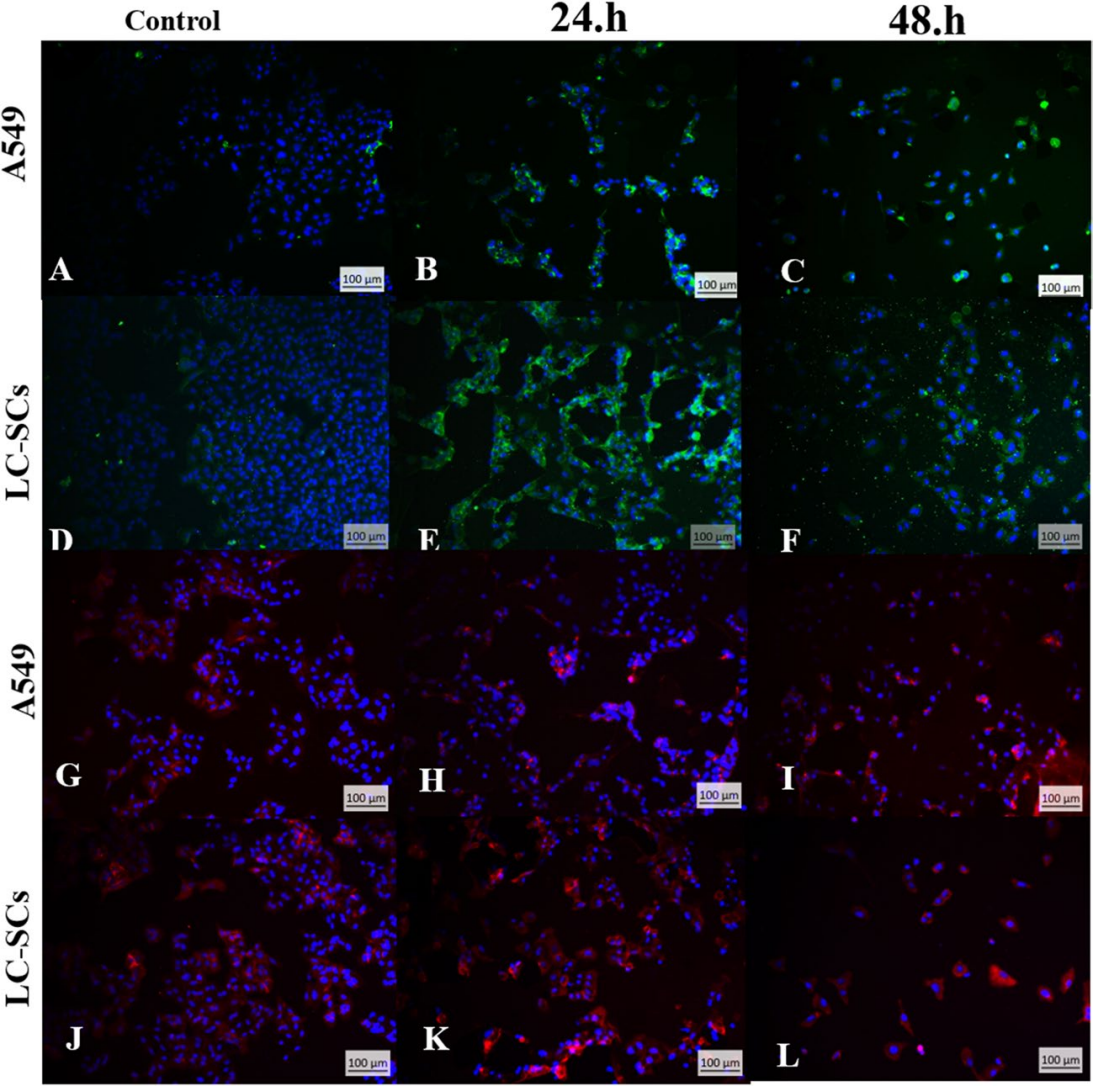
Immunocytochemistry analysis of caspase-3 and E-cadherin expression. BA increased the expression of caspase-3 and E-cadherin. After treatment with BA for 24 and 48 h, apoptosis in A549 cells and LC-SCs was assessed immunocytochemically. **A**–**F**: caspase-3 expression in green;** G**–**L**: E-cadherin expression in red. Nuclei were labeled with DAPI (blue). (Scale bar = 100 µm)

LC-SCs exhibited high levels of caspase-3 and E-cadherin expression after treatment with BA for 24 and 48 h (Fig. 5). Apoptosis was determined by labeling cells with anti-caspase-3 to assess the expression of caspase-3 in cells (Fig. 5A–F). The expression of caspase-3 in LC-SCs was observed to be higher (Fig. 5F). Cells were labeled with E-cadherin to assess the anti-metastatic effect of BA (Fig. 5G–L). The expression of E-cadherin in LC-SCs was observed to be higher (Fig. 5L).

## Discussion

Boric acid (BA) is a trace element naturally found in water, rocks, soil, and some foods. The major soluble form of boron in plasma is BA [24]. Several studies showed that BA affects the induction of apoptosis [25, 26]. Regarding pharmacologically relevant concentrations, BA demonstrated a dose-dependent reduction in the proliferation and invasion of cancer cell lines in varying in vitro amounts [27, 28]. We aimed to study boric acid as a cytotoxic agent due to its antiproliferative and apoptotic effects on different cancer cell lines. In in vitro studies, the effects of boric acid on cell proliferation and cytotoxicity were studied in long and short-term periods [23, 29–31]. In this study, we demonstrated the responses of LC-SCs to 48-h boric acid treatment. Our previous studies showed the antiproliferative effect of BA on colon cancer cells [23] and breast cancer stem cells [22]. We used the same BA doses (1–100 mM) used in these studies in this study. Since the RT-qPCR analysis was planned for the 48th hour and the lowest dose that reduced cell viability by more than 50% at the 48th hour was 50 mM, the only shared dose that could be used for 24 and 48 h in the experiments was determined as 50 mM. BA showed an antiproliferative effect in LC-SCs. The doses we applied were higher than the literature. However, since lower doses did not have an antiproliferative effect on cancer stem cells, we tried the doses we determined in our previous publications. We evaluated BA on cancer and cancer stem cells. The doses are high compared to the literature, and lower doses of BA alone may not be sufficient, so combined treatment may be needed. Additionally, while the doses used in this study stop the proliferation of cancer cells and cancer stem cells, evaluating them in terms of healthy cells is necessary. Perhaps these doses may be toxic to healthy cells and tissues. While it kills cancer and cancer stem cells, it can cause damage to the liver and kidneys. Thus, it may mean a general toxic effect of BA.

In a study conducted similar to the millimolar doses in our study, an antiproliferative effect was observed after 24 h of incubation with millimolar BA concentrations (0.5–20 mM) given to human breast cancer cells. Still, there was no significant change in caspase-3 level [32]. In the current study, we observed that the proliferation of cells decreased while the level of caspase-3 increased. No significant effect was detected on Bcl-2 protein levels or cytochrome c release in cells treated with boric acid, and only minor changes in caspase-3 activity were observed [15]. Regulation of cell cycle and DNA repair processes is provided by the S-phase checkpoint to adapt to the effects of replication stress, especially in cancer cells [33]. CSCs increase DNA repair capacity by keeping longer checkpoint intervals in the cell cycle and thus respond to DNA damage more efficiently. It has also been shown that ROS levels are reduced in CSCs, thus protecting their genomes from DNA damage. Cell cycle checkpoints, proteins involved in DNA repair, and intracellular redox balance are biological targets in cancer treatment [14]. Suppression of the DNA damage response (DDR) and associated signaling cascade can increase DNA damage tolerance and thus prolong the survival of CSCs. However, various studies have indicated that up-regulation of DNA repair pathways is important in CSCs due to their genomic instability. High levels of cell cycle checkpoint kinases and DNA repair proteins provide CSCs with robust armor against genotoxic therapy [14]. The expression levels of BRCA1, BRCA2, RAD51, KU70/80, ATM, and XRCC4 were investigated with molecular RT-qPCR. In our previous study, we studied the effect of BA on DNA DSB genes in breast cancer cells isolated from MCF-7. While BRCA1 and BRCA2 expression levels of breast cancer stem cells to which we applied the same dose increased in terms of DNA DSB repair, in the current study, it was observed that BRCA1 and BRCA2 levels decreased, but there was no significant change. Again, in the same study, the expression of ATM (*p* < 0.001), RAD51 (*p* < 0.001), and KU70 (*p* < 0.001) downregulated in dose-treated BC-SCs (*p* < 0.001).

Interestingly, in the current study, only the ATM (*p* < 0.001) expression increased approximately 30-fold. The application of BA was shown to be more effective in LC-SCs by increasing ATM expression. Suppression of the DNA damage response (DDR) and its associated signaling cascade can increase DNA damage tolerance and prolong the survival of CSCs. CSCs have been reported to be important for up-regulating DNA repair pathways to eliminate the adverse effects of genomic instability. Upregulation of DNA repair proteins and cell cycle checkpoint kinases protects against genotoxic treatment to CSCs [14]. Therefore, the downregulation of DNA DSB repair genes we obtained in the study may indicate that we are one step closer to treatment. The 30-fold increase in ATM may result from the induction of the MYBL2 transcription factor. However, the fact that other genes of DSB are not up-regulated can be considered harmful in terms of CSC survival. After all, if other genes were up-regulated, CSCs could continue to increase. We can say that BA is an important molecule in LC-SC therapy targeting. Caspase-3 and E-cadherin expression at the protein level was determined using immunofluorescence. LC-SCs treated with BA showed a significant increase in caspase-3 and E-cadherin expression. Therefore, BA could inhibit the DSB repair of lung cancer stem cells by increasing caspase-3 and E-cadherin. While transcriptional responses in physiological or pathological processes are measured by qPCR analysis, the presence or localization of relevant molecules in the tissue is determined by immunocytochemical analyses. Discrepancies observed between qPCR and immunocytochemical analyses may arise from differences in the transcriptional stage. Additionally, the loss of E-cadherin expression is expected for the active occurrence of epithelial-mesenchymal transition (EMT) in the tumor. The increase in E-cadherin expression after treatment with BA, as observed in immunofluorescence findings, suggests that BA may also prevent epithelial-mesenchymal transition.

In this study, we aimed to observe the impact of BA on the expression of DNA double-strand break (DSB) repair genes in A549 cancer stem cells. Boric acid affects the DNA DSB repair features of CSCs. Little is known about the relationship between lung cancer and DNA DSB repair. According to the results of our literature research, this is the first study to examine the effect of BA on DNA DSB repair of lung cancer stem cells. The study has some limitations. The results of our study may provide new insights into DNA DSB in lung cancer stem cells, but it is still unclear how CSCs and other cells found in the tumor microenvironment function in cancer invasion and migration. We could not examine the BA effect in vivo regarding its function on DDR. Extensive studies have revealed that BRCA proteins bind and interact with several regulatory proteins. The mechanism underlying these effects may be related to TGF-β, PI3K/Akt, and Wnt signaling pathway activation. It would be interesting to explore this aspect. Future studies need further to confirm the role of regulatory networks and signaling pathways.

Additionally, the limitations of this study are that the cells in the tumor microenvironment are not studied together, and advanced molecular techniques cannot be performed. This study is preclinical and also preliminary. Whether the dose determined in cell culture can be provided in plasma in a living organism or whether this dose negatively affects other healthy cells or systems is a situation that can only be determined through animal experiments and subsequent clinical trials. Ensuring the presence of the drug in the lungs at determined concentrations is also among the goals during drug development studies. The aim is to investigate whether BA affects gene expression related to the DNA repair mechanism in cancer stem cells and a preliminary study for now. However, our findings are a guide for further studies. Of course, the effects on the healthy lung cell line, as well as the cancer cell line, could also be examined. We could not perform Western Blot, Comet Assay analysis, clonogenic assay, and expression of phosphorylated H2A histone family member X (γH2AX). We will consider this analysis in our future studies. Herein, we focused on comparing the expression levels of the BRCA1, BRCA2, ATM, RAD51, KU70, KU80, and XRCC4 genes responsible for DSB HR repair using RT-qPCR. The findings of this study may provide new information on targeting proteins in the DSB repair pathway of lung CSC, evaluating BA for potential therapeutic application in cancer stem cell-targeted therapy.

## Data Availability

The datasets generated during and/or analyzed during the current study are presented in this paper.
